# Lesion network mapping of eye-opening apraxia

**DOI:** 10.1093/braincomms/fcad288

**Published:** 2023-10-27

**Authors:** Pardis Zarifkar, Nicholas A Shaff, Vardan Nersesjan, Andrew R Mayer, Sephira Ryman, Daniel Kondziella

**Affiliations:** Department of Neurology, Rigshospitalet, Copenhagen University Hospital, 2100 Copenhagen, Denmark; Mind Research Network, Albuquerque, NM 87131, USA; Department of Neurology, Rigshospitalet, Copenhagen University Hospital, 2100 Copenhagen, Denmark; Copenhagen Research Center for Mental Health—CORE, Copenhagen University Hospital, 2900 Copenhagen, Denmark; Mind Research Network, Albuquerque, NM 87131, USA; Mind Research Network, Albuquerque, NM 87131, USA; Department of Neurology, Rigshospitalet, Copenhagen University Hospital, 2100 Copenhagen, Denmark; Department of Clinical Medicine, University of Copenhagen, 1172 Copenhagen, Denmark

**Keywords:** aphasia, eye-opening apraxia, fMRI, hemiballismus, lesion network mapping

## Abstract

Apraxia of eyelid opening (or eye-opening apraxia) is characterized by the inability to voluntarily open the eyes because of impaired supranuclear control. Here, we examined the neural substrates implicated in eye-opening apraxia through lesion network mapping. We analysed brain lesions from 27 eye-opening apraxia stroke patients and compared them with lesions from 20 aphasia and 45 hemiballismus patients serving as controls. Lesions were mapped onto a standard brain atlas using resting-state functional MRI data derived from 966 healthy adults in the Harvard Dataverse. Our analyses revealed that most eye-opening apraxia-associated lesions occurred in the right hemisphere, with subcortical or mixed cortical/subcortical involvement. Despite their anatomical heterogeneity, these lesions functionally converged on the bilateral dorsal anterior and posterior insula. The functional connectivity map for eye-opening apraxia was distinct from those for aphasia and hemiballismus. Hemiballismus lesions predominantly mapped onto the putamen, particularly the posterolateral region, while aphasia lesions were localized to language-processing regions, primarily within the frontal operculum. In summary, in patients with eye-opening apraxia, disruptions in the dorsal anterior and posterior insula may compromise their capacity to initiate the appropriate eyelid-opening response to relevant interoceptive and exteroceptive stimuli, implicating a complex interplay between salience detection and motor execution.

## Teaser

Using lesion network mapping, Zarifkar *et al.* report that eye-opening apraxia is uniquely associated with the dorsal anterior and posterior insula. The study suggests a complex interplay between salience detection and motor execution in the initiation of appropriate eyelid-opening responses, with neural substrates distinct from other common stroke-related syndromes.

## Graphical Abstract

**Graphical Abstract:**
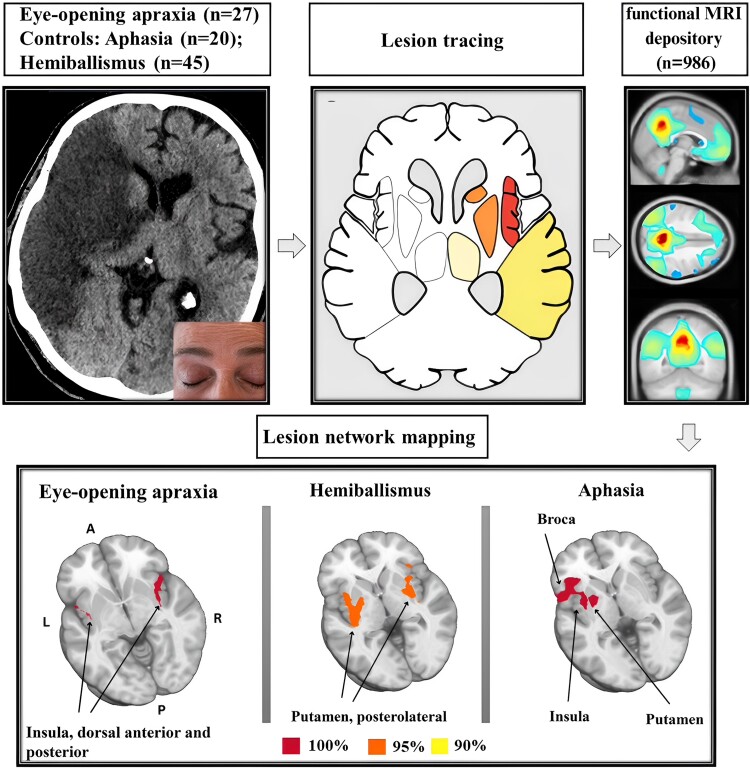

## Introduction

The voluntary control of eyelid opening and closure involves the coordinated activity of various central and peripheral nervous system structures and ocular muscles.^1,2^ Eyelid movement requires the reciprocal activation of orbicularis oculi and levator palpebrae superioris muscles, with Müller’s tarsal muscle assisting in eyelid elevation.^3^ While the infranuclear mechanisms of eyelid control and associated disorders are rather well understood, the supranuclear mechanisms of voluntary eyelid control, including the key brain networks, remain unclear and warrant further investigation.

Apraxia of eyelid opening (or eye-opening apraxia, EOA) is a non-paralytic disorder that disrupts the voluntary elevation of the eyelid.^2^ This term is somewhat controversial because it deviates from the standard definition of apraxia, which refers to neurological conditions affecting learned motor task.^2,4^ Despite normal eye movements and preserved blinking reflexes, individuals with EOA struggle to voluntarily open their eyelids.^1,5^ The pathophysiology involves excessive suppression of the levator palpebrae superioris muscle, delaying eyelid elevation or prolongation of orbicularis oculi muscle activity, extending eyelid closure.^6-8^ Subclinical orbicularis oculi activity can be identified only through electromyography, setting it apart from manifest blepharospasm.^8^ Causes include neurodegenerative processes, traumatic brain injury and focal lesions affecting eyelid control neural pathways.^5,9-15^ Despite affecting 4.7–6% of stroke patients and with a general population prevalence of 59 per million, EOA’s neural basis remains unknown.^3,10,15^ Existing research indicates right hemisphere dominance in cortical eyelid control,^3,5-16^ and the insular cortex, integrating interoceptive and exteroceptive signals, is thought to facilitate transitions between eye opening and eye closure.^16-22^

Lesion network mapping uses resting-state functional magnetic resonance imaging (rs-fMRI) data to identify functionally connected brain regions affected by focal lesions, providing insight into the network substrates of different neurological disorders.^23^ In this study, we applied lesion network mapping analysis in a diverse cohort of EOA stroke patients. Sensitivity and specificity analyses were conducted to ensure the robustness of our findings, and two neurological control conditions, i.e. aphasia and hemiballismus (by which we mean hemiballismus and/or haemichorea), served as reliability tests and to independently replicate previous study results.^23,24^ We hypothesized that, compared with control conditions, lesion network mapping would reveal distinct brain networks implicated in EOA.

## Materials and methods

### Clinical stroke cohorts and systemic literature reviews

**Figure 1:**
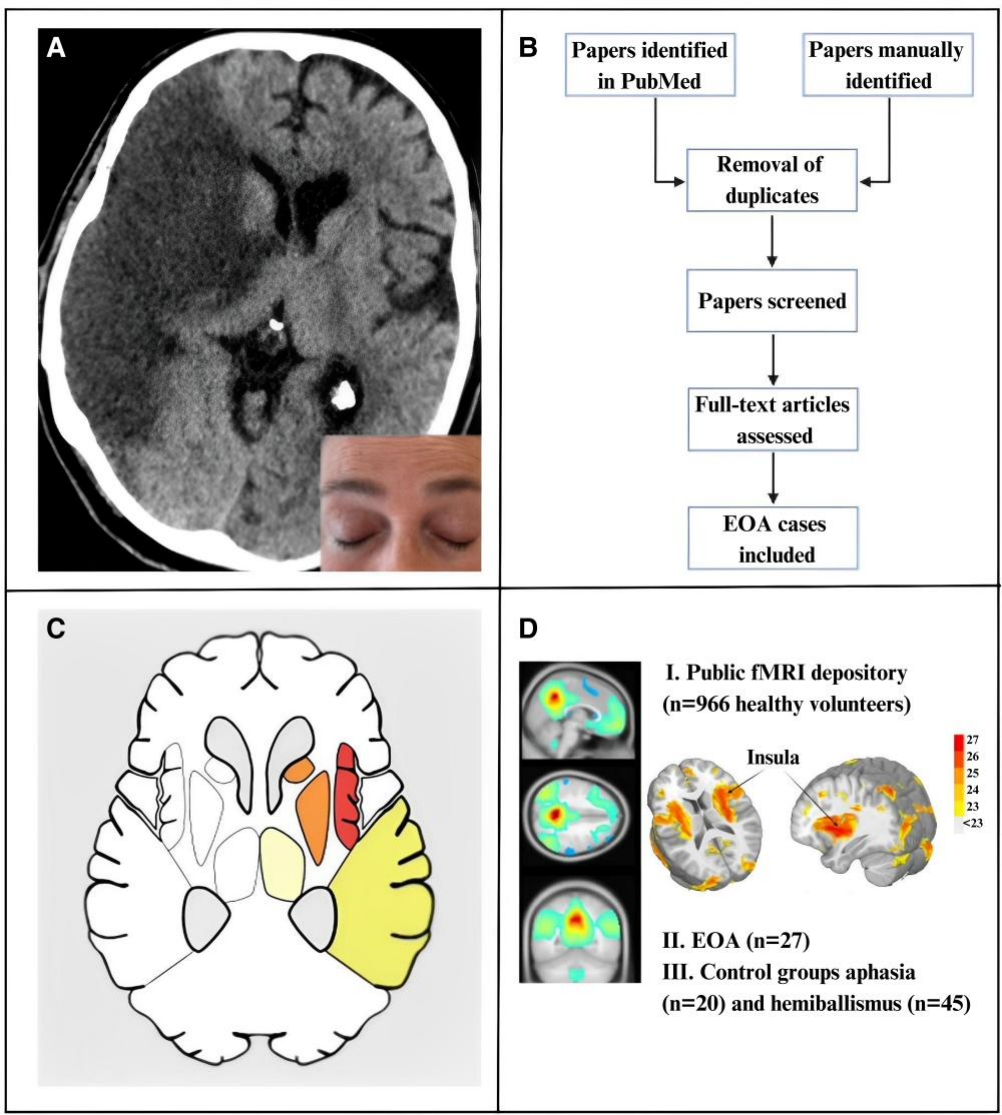
**Overview of EOA study design and lesion network mapping methodology.** (**A**) EOA is the non-paralytic inability to open the eyes in the absence of visible contraction of the orbicularis oculi muscle (insert; note contraction of the frontalis muscle). We identified EOA in six (6%) of 99 consecutive patients with anterior circulation stroke admitted for endovascular thrombectomy. (**B**) Following a systematic PubMed literature review, we included 21 further EOA cases. (**C**) Lesion network mapping begins by identifying brain lesions on CT or MRI in individual patients who share a similar neurological symptom (here, EOA), so lesions from all 27 patients were manually traced and mapped as 2D masks onto a standard brain atlas. (**D**) Utilizing a publicly available rs-fMRI database of healthy volunteers (I), the human connectome was then leveraged to identify brain networks associated with EOA (*n* total = 27, II). Stroke cases with aphasia (*n* = 20 of 99 consecutive patients with anterior circulation stroke admitted for endovascular thrombectomy) and hemiballismus (*n* = 45, identified from a systemic literature search), respectively, but EOA, served as controls (III). EOA, eye-opening apraxia; fMRI, functional magnetic resonance imaging.

The study methodology and rationale, depicted in Fig. 1, involved the prospective enrolment of adult patients with anterior circulation stroke undergoing endovascular thrombectomy over a 9-month period at a tertiary care centre, as previously reported.^10^ Exclusion criteria included reduced consciousness, lack of voluntary responses or spontaneous motor activity suggesting awareness, sedation lasting >48 h, pre-stroke major ophthalmological disorders and acute endovascular thrombectomy for posterior circulation strokes. The latter group was excluded to prevent confounding deficits from infranuclear oculomotor dysfunction. We rigorously assessed 99 stroke patients for eyelid motility, pupillary function and stroke neuroimaging to identify cases of EOA (detailed in Nersesjan *et al*.^10^). For specificity analyses, we also enrolled acute anterior circulation stroke patients with aphasia but no EOA from the same cohort (Fig. 1).

In addition, a comprehensive literature review was independently conducted by two investigators (P.Z. and D.K.) to identify additional EOA cases. Briefly, following a systematic PubMed literature review from inception until 30 May 2021, we searched articles in English, German and Japanese, using PRISMA guidelines, to identify further EOA cases. The literature search was supervised by the library service of the University of Copenhagen. Search terms included ‘eyes’, ‘eyelid’, ‘eye opening’, ‘apraxia’, ‘ptosis, ‘blepharoptosis’, ‘stroke’, ‘infarction’ and ‘haemorrhage’. Abstract and titles were screened first, followed by evaluation of full texts. Reference lists were searched manually to identify further eligible publications, and papers were cross-referenced using the ‘cited by’ function in PubMed. Inclusion criteria were EOA due to ischaemic/haemorrhagic focal lesions with available brain CT or MRI of sufficient quality to enable lesion tracing. The study population included patients of all ages and sexes.

For further specificity analyses, a third control cohort of stroke cases with hemiballismus (and no EOA) was collected using a literature review of cases published after 2014 (the year in which a previous hemiballismus lesion network mapping study^24^ based on a similar literature review had been published).

### Lesion network mapping

Our previous work focused on the prevalence and anatomical distribution of stroke lesions causing EOA.^10^ Here, we extend this understanding through a lesion network mapping analysis, investigating the functional connections of these and additional lesions identified in literature, to identify the common neural substrate of supranuclear eyelid control. We used rs-fMRI data from a cohort of 966 healthy individuals in the Harvard Dataverse^25^ to calculate functional connectivity between each lesion and all other voxels in the brain.^23^ Lesion tracing, pre-processing and resting-state pipelines adhered to previously published methods, ensuring methodological consistency.^23,26-28^ Lesions were traced using 3D Slicer version 49,^29^ and pre-processing and resting-state pipelines were conducted in SPM12^30^ and AFNI.^27^

#### Lesion tracing and 2D lesion masks

Lesion masks for patients with EOA and control conditions (hemiballismus and aphasia) were manually traced onto an MNI152 atlas using neuroanatomical landmarks. One investigator (P.Z.) performed the initial tracing, and a second investigator (D.K.) independently checked the work.

#### Pre-processing

Standard pre-processing procedures were followed for both structural and rs-fMRI data. For structural data, T_1_-weighted MRI images were pre-processed by uniformizing, skull stripping and normalizing to the MNI-152 template. Tissue segmentations were calculated on raw T_1_-weighted data, grey matter, white matter and binarized CSF probability files (0.6 probability). rs-fMRI data were pre-processed in the following order: slice-timing correction, motion correction using six motion regressors and derivatives, co-registration to the T_1_-weighted structural image and normalization to MNI space. Afterward, the data were spatially smoothed using a Gaussian kernel with a full width at half maximum of 6 mm. Next, band-pass filtering (0.01–0.1 Hz) was applied to the rs-fMRI data to reduce the influence of physiological noise. Finally, nuisance regression was performed using average white matter time series, average CSF time series and global signal as regressors.

#### Seed-to-voxel analyses

The average time series for each lesion mask served as a seed for correlation analyses with all remaining voxels in the residual file. Fisher *z*-transformations were applied to functional connectivity correlation maps, which were then thresholded at a conservative *t*-score threshold of *T* = 9.^31^ After thresholding, lesion-binarized files were summed and clustered using AFNI’s 3Dmerge (radius of the merging neighbourhood = 3.46 and volume multiplier = 200). The resulting lesion masks were analysed using multiple atlases (Brodmann, Harvard-Oxford cortical, XTRACT and Johns Hopkins University White Matter atlases). The overlap between each surviving cluster in the lesion masks and the atlases was used to identify distinct brain networks that may not be apparent using pre-defined binary thresholds.

#### Specificity analyses

To determine whether the brain regions and networks implicated in EOA are distinct from those involved in other stroke-related conditions, we compared the functional connectivity patterns observed in EOA stroke patients with those from stroke patients presenting with aphasia or hemiballismus but without EOA. Aphasia, due to larger lesions and from our clinical cases, and hemiballismus, due to smaller lesions and drawn from retrospective studies, provide contrasting lesion characteristics and network involvements compared with the EOA network. Our analysis first involved comparing clinical and anatomical lesion locations, followed by a repetition of lesion network mapping analyses for both hemiballismus and aphasia cohorts. By comparing the resultant functional connectivity maps at various overlapping thresholds (100%, 95% and 90%), we aimed to reduce the risk of false positive or negative results, further establishing the specificity of the identified neural substrates to EOA.

#### Sensitivity analyses

The sensitivity analyses aimed to evaluate the robustness of the identified brain regions and networks associated with EOA. To ensure that the findings were not influenced by potential confounding factors, such as differences in sample size, lesion location or methodology, we performed subgroup analyses (as outlined below) and comparisons with previous studies.

##### Subgroup analyses

We conducted subgroup analyses to assess the reliability and reproducibility of the EOA findings. The EOA lesions were randomly divided into two subgroups, and lesion network mapping analyses were performed separately for each subgroup. The functional connectivity maps between the two subgroups were compared to evaluate the consistency of the identified brain regions and networks associated with EOA. Specifically, we utilized the same clinical and literature-derived EOA lesions, dividing them into two groups: Group 1 (G1, *n* = 13) and Group 2 (G2, *n* = 14). The even- and odd-numbered volumes were treated as two separate data sets, with lesion network mapping analyses replicated for each data set. The resultant overlap maps from each data set were compared qualitatively through visual inspection and overlapping binary map files to assess consistency.

##### Comparison with previous studies

We conducted comparisons with previous studies to assess the general reliability of the lesion network mapping method, as well as its consistency and reproducibility across independent investigators and studies. We compared our findings with previous lesion network mapping studies of aphasia^23^ and hemiballismus,^24^ along with existing knowledge on EOA, in order to independently validate the networks involved in the control conditions and EOA.

### Statistical analysis

For baseline characteristics, we used descriptive statistics with continuous variables presented as median values with ranges or mean values with standard deviations, and categorical variables presented as frequencies and percentages. Inferential tests or qualitative tests were used to compare groups as appropriate (including chi-squared, Mann–Whitney *U* and Kruskal–Wallis tests). The significance level was set at 0.05, and all tests were performed using R (R 3.6.1, R Development Core Team, Vienna, Austria).^32^

### Ethics approval

The study was approved by the Ethics Committee of the Capital Region of Denmark (#19003059). The need for written consent was waived due to the retrospective nature of the study. Written informed consent was obtained for the publication of the clinical photograph in Fig. 1.

## Results

### Clinical characteristics and anatomic lesion distribution

**Figure 2:**
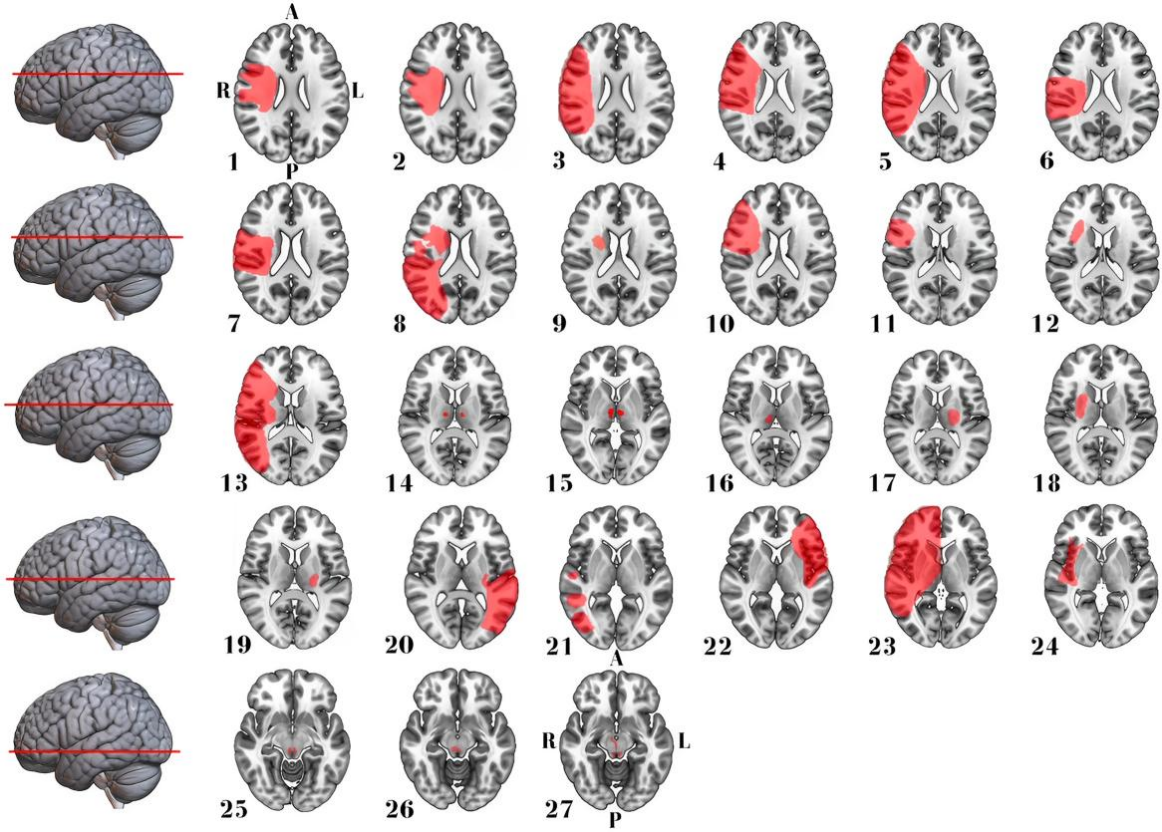
**Stroke lesion distribution in patients with EOA.** This figure presents 2D maps of ischaemic stroke or haemorrhage lesions that led to EOA in 27 patients (52% women) with a median age of 70 years. Each map represents a separate patient and is presented in radiological convention. Most lesions were in the right hemisphere and mixed cortical/subcortical involving the fronto-temporo-parietal lobes, thalamus or basal ganglia. Directional abbreviations indicate brain orientation: A, anterior; L, left; P, posterior; R, right.

**Figure 3:**
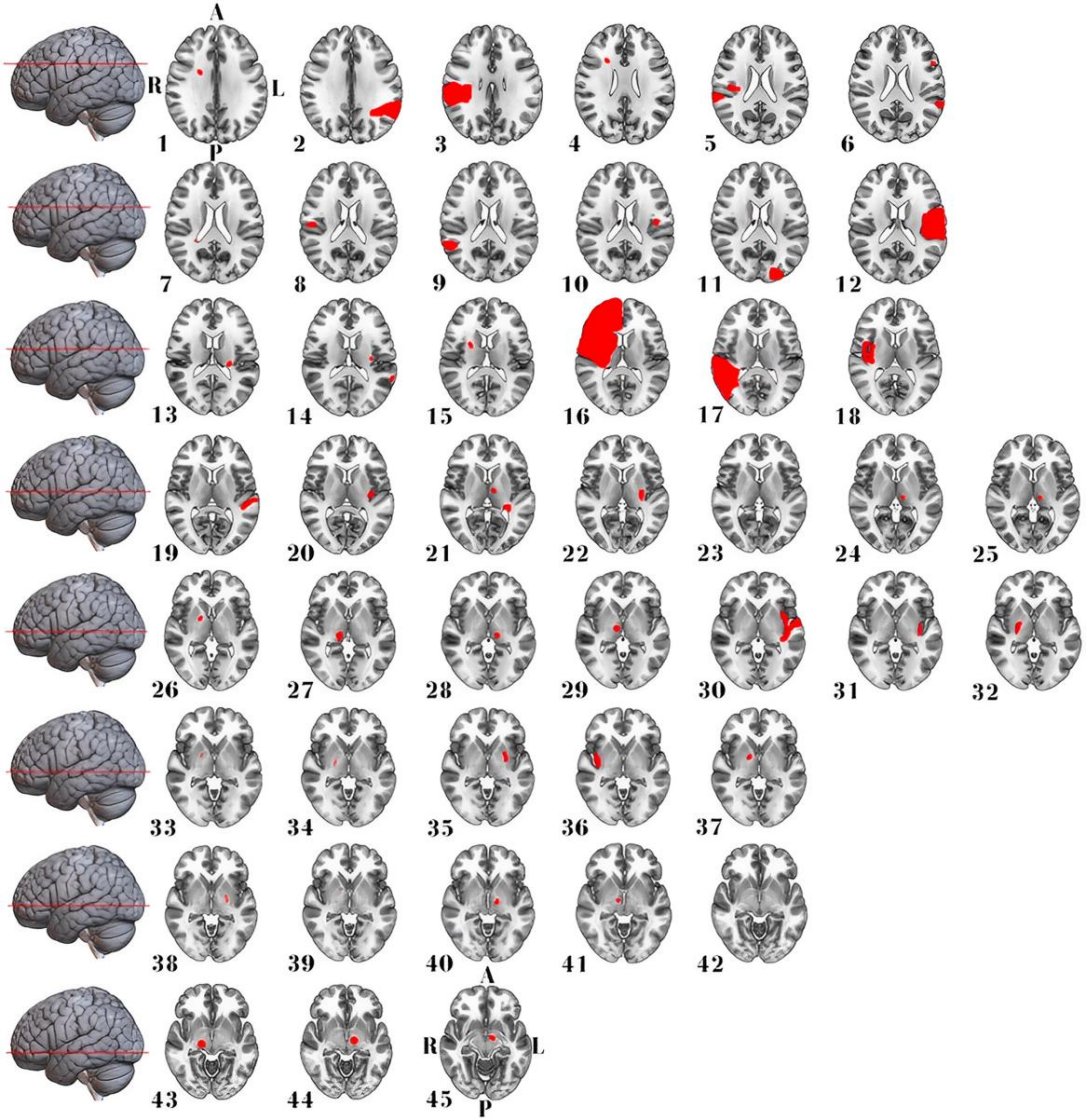
**Stroke lesion distribution in patients with hemiballismus.** This figure displays 2D maps of traced ischaemic stroke or haemorrhage lesions in radiological convention, each representing a separate patient. Lesions led to acute hemiballismus in 45 patients (56% women, median age of 72 years). Most lesions were in the right hemisphere and isolated subcortical. A, anterior; L, left; P, posterior; R, right.

**Figure 4:**
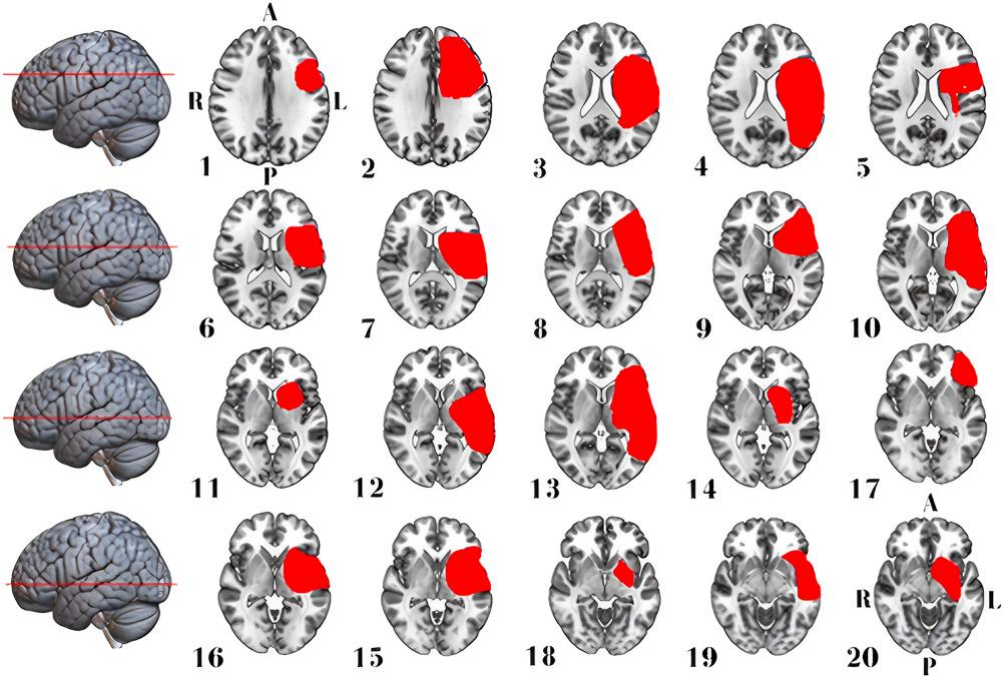
**Stroke lesion distribution in patients with aphasia.** This figure displays 2D maps of traced ischaemic stroke or haemorrhage lesions in radiological convention, each representing a separate patient. Lesions led to aphasia in 20 patients (65% women, median age of 73 years). All lesions were in the left hemisphere, and most were mixed cortical/subcortical. Directional abbreviations indicate brain orientation: A, anterior; L, left; P, posterior; R, right.

We included 27 EOA cases (median age 70, range 46–91 years; 14 women) resulting from ischaemic or haemorrhagic stroke. This cohort comprised six cases from our institution (clinical details are described in Nersesjan *et al*.^10^) and 21 identified through a systematic literature review (Fig. 1). Age and sex distributions did not differ between EOA cases and control stroke cases without EOA (*n* = 20 with aphasia, *n* = 45 with hemiballismus; Table 1). EOA and aphasia lesions predominantly involved the right (70%) or left (100%) hemispheres, respectively, while hemiballismus lesions were distributed evenly across both hemispheres (≍50% each). Most EOA (70%) and aphasia (70%) lesions were mixed cortical/subcortical, while hemiballismus lesions were primarily isolated subcortical (62%). Over 90% of hemispheric EOA lesions involved the fronto-temporo-parietal lobes, thalamus or basal ganglia. Detailed clinical and anatomic information for EOA and control lesions is presented in Figs 2–4 and in Supplementary Tables 1 and 2.

**Table 1:** Clinical characteristics

	Eye-opening apraxia (*n* = 27)	Hemiballismus (*n* = 45)	Aphasia (*n* = 20)	Significant *P*-values^a^
Demographics				
Women	14 (52%)	25 (56%)	13 (65%)	-
Median age (range), years	70 (46–91)	72 (23–93)	73 (33–92)	-
Lateralization				
Right hemisphere	19 (70%)	23 (51%)	-	-
Left hemisphere	3 (11%)	22 (49%)	100 (%)	< 0.0001^a^
Midline structures^b^	5 (19%)	-	-	-
Localization				
Isolated cortical	-	9 (20%)	1 (5)	-
Isolated subcortical	8 (30%)	28 (62%)	5 (25%)	0.002^a^
Cortical-subcortical^c^	19 (70%)	8 (18%)	14 (70%)	0.02^a^

^a^See Materials and methods. ^b^Includes paramedian thalamic, thalamus, midbrain tectum and tegmentum. ^c^Includes brainstem for eyelid-opening apraxia.

### Lesion network mapping of EOA

**Figure 5:**
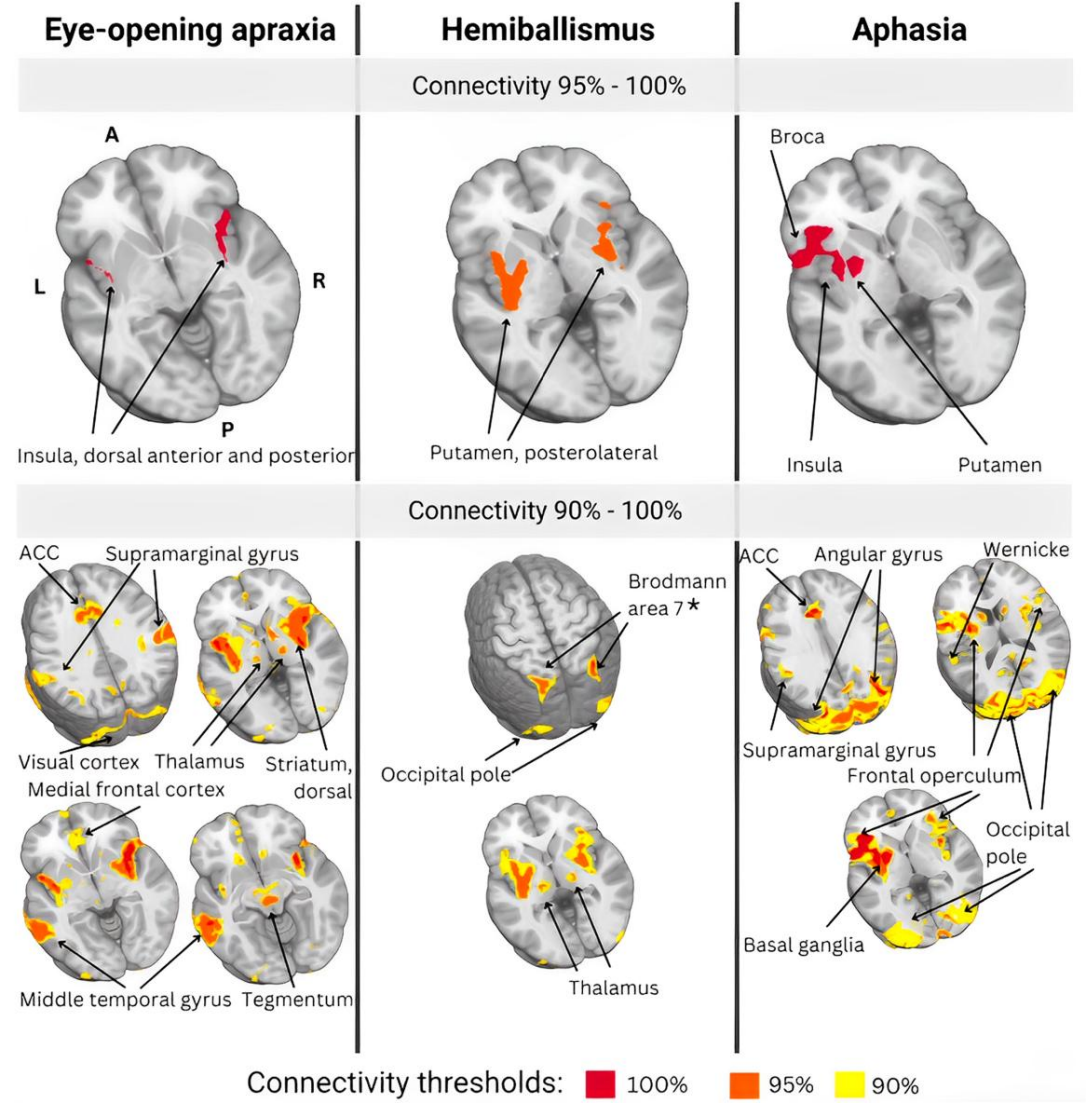
**Comparative lesion network overlaps: EOA, hemiballismus, and aphasia mapping.** The top panel displays areas of highest overlap (95–100%) between anatomic lesions and functional mapping results, while the bottom panel depicts areas of partial overlap (90%–100%). EOA: in patients with EOA, all (100%) anatomic lesions mapped onto the bilateral insula, particularly dorsal anterior and posterior portions. In addition, 95% of lesions mapped onto the middle temporal gyrus, and over 90% onto the anterior cingulate cortex, post-central gyrus, thalamus, superior parietal lobule, medial prefrontal cortex, supramarginal gyrus, dorsal striatum, visual cortex, right posterior cerebellar hemisphere and tegmentum (oculomotor nucleus). Overall, lesion network mapping reveals the importance of the insula as a key node of the EOA network. Hemiballismus: in patients with hemiballismus, over 95% of lesions mapped onto the bilateral putamen, particularly the posterolateral portion. Over 90% of lesions also mapped onto the posterior parietal cortex and precuneus (Brodmann area 7), precentral gyrus (particularly Brodmann Area 6), thalamus, supramarginal gyrus, anterior cingulate cortex and occipital pole. Aphasia: in patients with aphasia, all lesions mapped onto brain regions involved in language processing, specifically the left frontal operculum, particularly Broca’s area, left insula, supplemental motor cortex, left putamen and occipital pole, including primary visual and visual associative cortex, and lingual gyrus. Functional maps are presented in neurologic convention and directional abbreviations indicate brain orientation: A, anterior; ACC, anterior cingulate cortex; L, left; P, posterior; R, right. *Part of the posterior parietal lobe.

Lesion network mapping results are depicted in Fig. 5. Lesion network mapping showed that all (100%) EOA lesions functionally connected to the bilateral insula, particularly the dorsal anterior and posterior regions. At lower overlap thresholds, 95% connected to the middle temporal gyrus, and 90–95% mapped onto the anterior cingulate cortex, post-central gyrus, thalamus, superior parietal lobule, medial prefrontal cortex supramarginal gyrus, dorsal striatum, visual cortex, right posterior cerebellar hemisphere and dorsal tegmentum. Notably, most connectivity results were bilateral; i.e., functionally connected regions were distributed almost evenly across both hemispheres and midline structures, contrasting with the predominantly right hemisphere anatomical locations of the stroke lesions.

### Subgroup analyses

**Figure 6:**
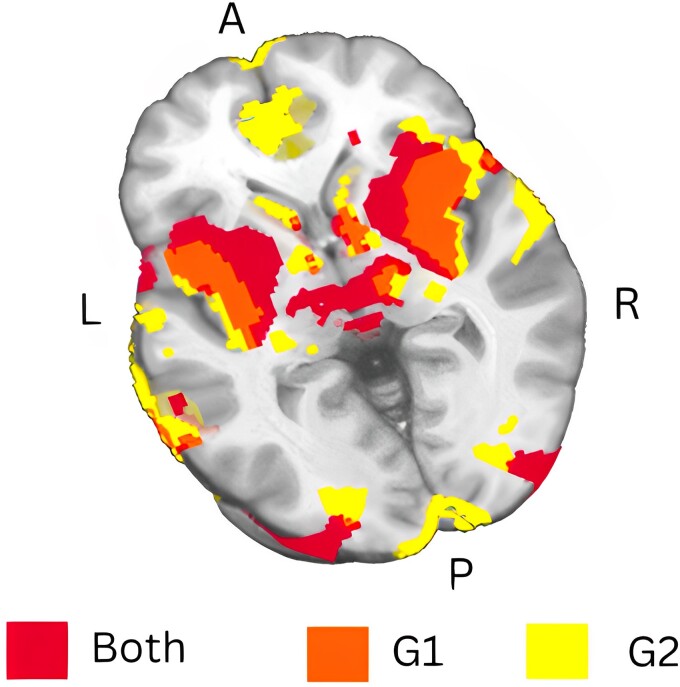
**Consistency in lesion connectivity patterns of EOA across subgroups.** This figure shows the results of a sensitivity analysis for two subgroups of EOA patients: G1 (*n* = 14) and G2 (*n* = 13). Both groups display a consistent lesion network connectivity pattern, visualized at an overlap threshold of 90%. Functional maps are presented in neurologic convention and directional abbreviations indicate brain orientation: A, anterior; ACC, anterior cingulate cortex; L, left; P, posterior; R, right.

Subgroup analyses of EOA lesions [*n* = 14 (G1), *n* = 13 (G2)] demonstrated a consistent pattern of lesion connectivity (Fig. 6) compared with each other and the combined analysis. Both G1 and G2 connected to the bilateral insula, middle temporal gyrus and supramarginal gyrus. Furthermore, all G1 lesions connected to the visual cortex, anterior and posterior cingulate gyri and tegmentum, while G2 lesions connected to the thalamus. At the 90% threshold, both G1 and G2 lesions connected to these regions.

### Distinct network maps for lesion network mapping of hemiballismus and aphasia

Lesion network mapping of the control conditions hemiballismus and aphasia revealed distinct brain networks when compared with each other and EOA.

For hemiballismus, 95% of lesions were connected to the bilateral putamen, specifically the posterolateral putamen. These findings demonstrate specificity compared with EOA and corroborate the results of a separate hemiballismus study conducted on a different patient cohort by other investigators.^24^ At a 90% overlap threshold, lesions also connected to brain regions involved in motor control, planning, somatosensory processing and visuospatial processing, including the parietal cortex and precuneus (Brodmann area 7), precentral gyrus (Brodmann area 6), thalamus, supramarginal gyrus, anterior cingulate cortex and occipital pole.

In patients with aphasia and no EOA, lesions were connected to brain regions primarily involved in language processing, predominantly in the left hemisphere, contrasting with EOA. Specific areas included the frontal operculum, encompassing Broca's area and the anterior insula, supplemental motor cortex, putamen, and occipital fusiform and associative visual cortices. These findings align with a previous lesion network mapping study on expressive subcortical aphasia, where all lesions were functionally connected to Broca’s area.^23^ Lesion network mapping results can be seen in Fig. 5.

## Discussion

Here, we showed that lesions associated with EOA are distributed across multiple brain regions but converge within a shared neural network. Our results suggest that EOA is a network disorder impacting a key network node in the bilateral dorsal anterior and posterior insula. Although the lesions were anatomically right lateralized, consistent with our previous observations,^10^ the functionally connected regions in the broader EOA network were distributed across both hemispheres and midline structures, contrasting with previous studies suggesting right hemisphere dominance for voluntary lid control.^1,3,10^ Distinctly different connectivity patterns were identified for control conditions, with bilateral putamen involvement in hemiballismus and language-processing areas in aphasia.

### The EOA network and its critical node, the insula

Lesion network mapping of EOA indicated that the insula governs the innate process of voluntary eyelid opening.^33^ The insula is responsible for mediating appropriate behavioural responses to salient stimuli by facilitating the detection, filtering and prioritization of relevant stimuli among multiple competing inputs.^18-20,33,34^ In the context of eyelid opening, our findings are consistent with prior imaging studies suggesting the insula functions as a switch between exteroceptive and interoceptive networks during eyelid opening and closing.^16,18-22,33,35-38^ Subregional specialization is observed in the insula from early development, with dorsal and ventral areas in the anterior and posterior insula having specific roles in body-centred and world-centred functions.^33^ The posterior insula integrates proprioceptive, visual and auditory stimuli, which are subsequently relayed to the anterior insula to transform objective representations of physiological changes into subjective experiences.^21,33-35,39-42^ The dorsal anterior insula, in cooperation with the anterior cingulate cortex, engages the motor system to generate appropriate control signals for the regulation of behaviour.^21,33-35,39-42^

Another significant node in the identified EOA network was the middle temporal gyrus. This structure, specifically of the left hemisphere, is involved in attention and visual processing during eyelid opening, particularly in response to visual motion stimuli.^43^ The broader EOA network identified in our study encompassed regions involved in perception and sensory processing (posterior insula, middle temporal gyrus, supramarginal gyrus and thalamus), attention, decision-making and response planning (anterior insula, anterior cingulate cortex, medial prefrontal cortex, superior parietal lobule and supplementary motor area), motor execution (dorsal tegmentum), and feedback processing and regulation (insula, dorsal striatum and the posterior cerebellar hemisphere). Prior research has demonstrated that electrical stimulation of medial frontal, temporal, parietal and occipital cortices, as well as the dorsal tegmentum, can induce eyelid opening.^3,44^ Interestingly, these brain regions are relevant not only in the context of acute EOA resulting from focal stroke lesions, as in our study, but also in the context of reduced eye blinking in patients with supranuclear palsy.^45^

In summary, our findings highlight the critical role of the insula and its connections within the EOA network. Disruptions in this network appear to compromise the ability of patients with EOA to initiate eyelid opening after appropriate interoceptive and exteroceptive stimuli.

### Networks for hemiballismus and aphasia differ from the EOA network

While lesion network mapping of EOA revealed in the importance of the insula, we observed involvement of the putamen bilaterally in hemiballismus and language-processing areas in aphasia.

In contrast to primarily right-centred EOA lesion, aphasia-related lesions were left-sided both anatomically and functionally. Lesions associated with aphasia are mapped onto brain regions that are key to various language-processing aspects, including speech production, syntax, semantics, phonological processing and auditory-visual information integration.^23,46^ These regions included the left frontal operculum, particularly Broca’s area, left insula, supplemental motor cortex, left putamen and occipital pole.

In contrast to EOA and aphasia, lesions associated with hemiballismus mapped onto separate brain regions and networks. Our study provides evidence supporting the central role of the putamen, particularly the posterolateral region, in the pathophysiology of hemiballismus. This subregion with the basal ganglia circuitry is implicated in the selection and execution of motor programs, as well as the integration of sensory and cognitive information during these processes.^47,48^ Damage to the putamen may disrupt the regulation of goal-directed movement and feedback control in hemiballismus.^47,48^ This finding is consistent with previous studies supporting the role of the putamen, caudate and subthalamic nucleus in hemiballismus.^47,48^ However, ours and a previous lesion network mapping study^24^ did not show an association between the subthalamic nucleus^49^ and stroke-induced hemiballismus lesions. In addition to the putamen, the broader hemiballismus network encompassed the parietal cortex and precuneus (Brodmann area 7), precentral gyrus (Brodmann area 6), thalamus, supramarginal gyrus, anterior cingulate cortex and occipital pole, indicating that hemiballismus may result from damage to a distributed network of brain regions engaged in motor control, movement planning and visuospatial and somatosensory integration.^47,48^

### Sensitivity, specificity and critical appraisal of the lesion network mapping methodology

We evaluated the robustness of the identified EOA network through comprehensive sensitivity analyses to adjust for confounding factors like sample size, lesion location or methodological discrepancies. We utilized a combined lesion data set derived from various sources, including anterior-circulation stroke patients from our institution and from the existing literature, with the intent to minimize biases associated with EOA aetiology, lesion location and clinical diagnosis. A two-stage process was used to increase the accuracy and reliability of the lesion mask tracing.

First, as no previous lesion network mapping study of EOA has been done, we conducted subgroup analyses to assess the sensitivity of the analyses in the absence of independent replication cohorts. The results revealed a consistent pattern of lesion connectivity centred on the bilateral dorsal anterior and posterior insula. This outcome confirmed the robustness of the identified brain regions, which remained consistent despite the separation of a substantial proportion of lesions from the analyses and the relatively low total number of 27 lesions. Second, specificity analyses were conducted to determine whether the cerebral regions and networks implicated in EOA were distinct from those involved in other stroke-related conditions.

We selected aphasia and hemiballismus as control conditions for their distinct network involvement and varied lesion characteristics. Aphasia, from larger lesions, impacts broad dominant cerebral hemisphere regions, while hemiballismus, from smaller lesions, involves basal ganglia-thalamo-cortical loops. These diverse conditions in our specificity analyses confirm the specificity of our EOA findings, regardless of lesion size or data source. Furthermore, to our knowledge, this is the first time that an independent team of investigators replicated previous lesion network mapping results,^23,24^ demonstrating that the lesion network mapping methodology yields consistent and reproducible results.

Given the large number of voxels involved in fMRI analyses, we applied a medium-conservative *t*-threshold to balance sensitivity and specificity in controlling the false positive rate, remaining consistent with previously reported thresholds.^24^ Distinct regions that may have been overlooked with pre-defined binarizing thresholds were identified using clustering approaches. However, consensus on *t*-thresholding, voxel-level and cluster-level thresholding is still lacking. We visually inspected lesion maps and mapped them onto multiple atlases, presenting varying results for 100%, 95% and 90% overlaps. These findings underscore the importance of developing robust standard calibration methods for lesion network mapping analyses and addressing lesion heterogeneity and numbers when selecting overlap thresholds.

## Conclusion

Future lesion network mapping research should investigate the generalizability of the results to posterior circulation aetiologies and validate results from cohorts like ours in individual fMRI patient studies. Future lesion network mapping research should validate results from cohorts like ours in individual fMRI patient studies. It remains to be seen how well networks identified from lesion network mapping in retrospective cohorts generalize to individual and prospectively investigated stroke patients. Challenges in conducting this validation include small sample sizes, inter-individual variability and technical and methodological limitations. Overcoming these challenges will necessitate multicentre studies to increase sample sizes, application of advanced neuroimaging analysis methods to account for inter-individual variability and the implementation of rigorous quality control measures.

Our results indicate that lesions associated with EOA are found in diverse brain areas but converge within a specific network characterized by connectivity to a key node involving the bilateral dorsal anterior and posterior insula. The EOA network includes various functional domains responsible for perception, sensory processing, attention, decision-making, response planning, motor execution and feedback processing and regulation. We also showed that this EOA network is different from the networks of other neurological conditions and that the lesion network mapping methodology is reliable and consistent.

In sum, we suggest that stroke patients with EOA are unable to initiate eyelid opening in response to interoceptive or exteroceptive stimuli because of disruptions within the EOA network, particularly in the dorsal anterior and posterior insula.

## Supplementary Material

fcad288_Supplementary_Data

## Data Availability

Lesion data that support lesion network mapping findings of this study are freely available in the manuscript and in the supplementary materials. Any additional data sets are available from the corresponding author upon reasonable request.
