# Supplementary material for: Lesion network mapping of eye-opening apraxia
Source: Brain Commun. 2023 Oct 27;5(6):fcad288. doi: 10.1093/braincomms/fcad288 (PMC10636562; doi:10.1093/braincomms/fcad288)
Supplement: fcad288_Supplementary_Data [file fcad288_supplementary_data.pdf]

Supplemental tables

Table S1. Twenty-seven stroke cases of eye-opening apraxia included in lesion-network-mapping analysis

**Source**	**N**	**Imaging**	**Location**	**Lesion location**	**Depiction in Figure 1**
Algoed et al. 1992[1]	1	CT	m	Rolandic artery territory (r)	11
Averbuch-Heller et al. 2002[2]	2	CT	m; m	Frontoparietal lobe (r); MCA territory (r)	6, 23
Averbuch-Heller et al. 1996[3]	1	CT	m	Frontoparietal lobe (r)	7
Ferro et al. 2021[4]	1	MRI	s	Paramedian thalamic (bilateral)	15
Hirose et al. 2010[5]	2	CT	s; s	Thalamus (r); globus pallidus/thalamus (l)	19, 16
Hsieh et al. 2014[6]	1	MRI	m	Parietotemporal lobe (r)	21
Johnston et al. 1989[7]	2	CT	m; m	MCA territory (r) x2	1, 2
Kaiboriboon et al. 2002[8]	1	MRI	m	MCA territory (l)	20
Kim et al. 2015[9]	1	MRI	b	Midbrain tectum (bilateral)	25
Lanczik et al. 2005[10]	1	MRI	m	MCA territory (r)	3
Lee et al. 2011[11]	1	MRI	s	ICA borderzone territory, frontal (r)	12
Lin et al. 2013[12]	1	CT	s	Putamen (r)	9
Mihaescu et al. 2000[13]	1	MRI	b	Midbrain tegmentum (bilateral)	26
Myint et al. 2008[14]	1	MRI	s	Paramedian thalamic (bilateral)	14
* Nersesjan et al. 2020[15]	6	MRI/CT	m x5; s x1	ICA territory (r) x3, MCA territory (r) x3	4, 5, 8, 13, 18, 24
Raibagkar et al. 2017[16]	1	MRI	b	Midbrain tegmentum (bilateral)	27
Sakajiri et al. 1995[17]	1	MRI	m	MCA territory (r)	10
Verghese et al. 1999[18]	1	CT	s	Putamen (l)	17
Waragai et al. 1996[19]	1	MRI	m	MCA territory (r)	22
B – brainstem, ICA – internal carotid artery, l – left, MCA – middle cerebral artery; m – mixed cortical/subcortical; r – right, s- subcortical, * six cases prospectively identified at the authors' institution					

Table S2. Forty-five stroke cases of hemiballismus included in lesion-network-mapping analysis

**Source**	**N**	**Imaging**	**Location**	**Lesion**	**Depiction in Figure 2**
Ahn and Kwon 2017 [20]*	1	MRI	s	Striatocapsular area (l)	22
Aiba et al. 2021 [21]	1	MRI	s	Striatum (l)	38
Bacchini et al. 2018 [22]	1	MRI	s	Thalamus (l)	23
Bembenek et al. 2015 [23]	1	MRI	s	Globus pallidus (r)	34
Bertamino et al. 2021 [24]	1	MRI	s	Medial thalamus (l)	25
Carbayo et al. 2020 [25]	4	MRI	m; c; c; c	Parietal and posterior frontal cortices, sparing the basal ganglia (r); insular, frontal, and postcentral gyrus (l); parietal and insular cortex, with complete sparing of the basal ganglia (r); temporal, and parietal cortex (l)	8, 12, 9, 19
Chen and Xu 2020 [26]	1	MRI	s	Globus pallidus (r)	33
Cotroneo et al. 2020 [27]	3	MRI	m; m; c	Frontoparietal cortical and subcortical region (r); frontoparietal and insular cortex (r); frontal-parietal-insular cortex (l)	5, 18, 6
Damani et al. 2015 [28]	1	MRI	s	Lentiform nucleus (l)	35
Disserol et al. 2020 [29]	1	MRI	m	Insular cortex and capsula extrema (r)	36
Ganapa et al. 2019 [30]	1	MRI	s	Thalamus and subthalamic nucleus (r)	27
Grassl et al. 2022 [31]	1	MRI	s	Subthalamic nucleus extending to the anterior thalamus (l)	45
Hernandez Fustes et al 2020 [32]	1	CT	m	Temporo-parietal lobe (r)	17
Hodde et al. 2017 [33]	1	CT	s	Thalamus (l)	42
Huang and Gho 2021 [34]	1	MRI	s	Centrum semiovale (r)	4
Kannepalli et al. 2016 [35]**	1	MRI	m	ICA territory with sparing of the thalamus and part of the lentiform nucleus (r)	16
Kato et al. 2021 [36]	1	MRI	c	Postcentral gryus and posterior part of insula (r)	3

Kurihara et al. 2021 [37]***	1	MRI	s	thalamic hemorrhage expanded to the subthalamic nucleus (r)	43
Li et al. [38]**	1	MRI	s	Dorsal thalamus and cerebral peduncle (l)	44
Maruyama et al. 2021 [39]	1	MRI	c	Insular cortex and parietal cortex (l)	20
Miller et al. 2018 [40]	1	MRI	s	Thalamus and adjacent to the atria of lateral ventricle (l)	21
Miura et al. 2017 [41]	1	CT	s	Thalamus (l)	24
Motolese et al. 2020 [42]	1	MRI	m	Temporo-parietal lobes and insular cortex (l)	14
Muguruma et al. 2019 [43]	1	MRI	m	Parasagittal frontal cortex and posterior horn of lateral ventricle (r)	7
Muhammad et al. 2019 [44]	1	CT	s	Parietal white matter (l)	10
Noda et al. 2015 [45]	1	MRI	s	Subthalamic nucleus (l)	40
Nohria et al. 2021 [46]	1	MRI	s	Cerebral peduncle (r)	41
Onder 2017 [47]	1	MRI	s	Posterior putaminal region (r)	13
Onder 2019 [48]	1	MRI	s	Centrum semiovale (r)	1
Qiu et al. 2018 [49]	1	MRI	s	Putamen (r)	15
Shahali et al. 2022 [50]	1	MRI	s	Thalamus (l)	28
Shiraiwa et al. 2020 [51]	3	MRI	s; s; s	Globus pallidus (r); globus pallidus (r); globus pallidus and putamen (r)	39, 26, 37
Shresta et al. [52]	1	MRI	c	Posterior parietal cortex (l)	2
Sikri et al. 2016 [53]	1	MRI	s	Thalamus (r)	29
Strauss et al. 2019 [54]	1	MRI	c	Parieto-occipital cortex (l)	11
Tatum et al. 2021 [55]	1	MRI	c	Temporal and insular cortex (l)	30
Tolla et al. 2020 [56]	1	MRI	s	Internal capsule, basal ganglia (l)	31
Ueta et al. 2021 [57]	1	MRI	s	Putamen (r)	32

ICA – internal carotid artery, l – left, MCA – middle cerebral artery; m – mixed cortical/subcortical; r – right, s- subcortical, * nocturnal hemiballismus, ** hemiballismus ipsilateral to the lesion, *** crossed hemiballismus (left upper, right lower limbs)

References

1. Algoed L, Janssens J, Vanhooren G. Apraxia of eyelid opening secondary to right frontal infarction. *Acta Neurologica Belgica*. 1992;92(4):228-233.
2. Averbuch-Heller L, Leigh RJ, Mermelstein V, Zagalsky L, Streifler JY. Ptosis in patients with hemispheric strokes. *Neurology*. 2002;58(4):620-624. doi:10.1212/WNL.58.4.620
3. Averbuch-Heller L, Stahl JS, Remler BF, Leigh RJ. Bilateral ptosis and upgaze palsy with right hemispheric lesions. *Annals of Neurology*. 1996;40(3):465-468. doi:10.1002/ana.410400320
4. Ferro D, Lopes C, Filipe JP, Guimarães J. Open the eyes to see the lesion: the hidden stroke. *Acta Neurologica Belgica 2021*. Published online January 12, 2021:1-2. doi:10.1007/S13760-020-01546-5
5. Hirose M, Mochizuki H, Honma M, Kobayashi T, Nishizawa M, Ugawa Y. Apraxia of lid opening due to a small lesion in basal ganglia: two case reports. *Journal of neurology, neurosurgery, and psychiatry*. 2010;81(12):1406-1407. doi:10.1136/JNNP.2009.184846
6. Hsieh CY, Sung PS, Hwang WJ. Transient blepharospasm, apraxia of eyelid opening, and hemidyskinesia following a right parietotemporal infarct. *Parkinsonism & related disorders*. 2014;20(9):1024-1026. doi:10.1016/J.PARKRELDIS.2014.05.012
7. Johnston JC, Rosenbaum DM, Picone CM, Grotta JC. Apraxia of eyelid opening secondary to right hemisphere infarction. *Annals of neurology*. 1989;25(6):622-624. doi:10.1002/ANA.410250615
8. Kaiboriboon K, Oliveira GR, Leira EC. Apraxia of eyelid opening secondary to a dominant hemispheric infarction [1]. *Journal of Neurology*. 2002;249(3):341-342. doi:10.1007/s004150200015
9. Kim DE, Kim SS, Kim SH, Kang KW, Nam TS, Lee SH. Midbrain infarction presenting with binocular elevation palsies and ptosis. *Neurological Sciences 2015 36:10*. 2015;36(10):1939-1941. doi:10.1007/S10072-015-2289-8
10. Lanczik O, Binder J, Lecei O, Schwarz S, Hennerici M. Open your eyes! *Neurocritical Care 2005 3:1*. 2005;3(1):61-62. doi:10.1385/NCC:3:1:061
11. Lee SS, Lee HS. Can subcortical infarction cause apraxia of eyelid opening? *Journal of Clinical Neuroscience*. 2011;18(10):1399-1400. doi:10.1016/J.JOCN.2010.12.055
12. Lin YH, Liou LM, Lai CL, Chang YP. Right putamen hemorrhage manifesting as apraxia of eyelid opening. *Neuropsychiatric disease and treatment*. 2013;9:1495-1497. doi:10.2147/NDT.S50974
13. Mihaescu M, Brillman J, Rothfus W. Midbrain ptosis caused by periaqueductal infarct following cardiac catheterization: early detection with diffusion-weighted imaging.

Journal of neuroimaging : official journal of the American Society of Neuroimaging. 2000;10(3):187-189. doi:10.1111/JON2000103187

14. Myint PK, Anderson KN, Antoun NM, Warburton EA. Eyelid apraxia associated with bilateral paramedian thalamic infarct. *Age and ageing*. 2008;37(3):343-344. doi:10.1093/AGEING/AFN016
15. Nersesjan V, Martens P, Truelsen T, Kondziella D. After stroke, apraxia of eyelid opening is associated with high mortality and right hemispheric infarction. *Journal of the neurological sciences*. 2020;418. doi:10.1016/J.JNS.2020.117145
16. Raibagkar P, Chavali R V., Kaplan TB, et al. Reverse Locked-In Syndrome. *Neurocritical care*. 2017;27(1):108. doi:10.1007/S12028-017-0391-X
17. Sakajiri KI, Matsubara N, Nakajima T, Fukuhara N, Bandoh M. A case of “apraxia of eyelid opening” secondary to right hemisphere infarction. Assessment of various symptoms of the eye and eyelid. *Clinical Neurology*. 1995;35(2):164-168.
18. Verghese J, Milling C, Rosenbaum DM. Ptosis, blepharospasm, and apraxia of eyelid opening secondary to putaminal hemorrhage. *Neurology*. 1999;53(3):652. doi:10.1212/WNL.53.3.652-A
19. Waragai M, Shinotoh H, Kaneko M, Hattori T. Difficulty in eye opening following left hemispheric infarction: Causative lesion and pathophysiology of abnormalities of the eye and eyelids movements. *Clinical Neurology*. 1996;36(4):577-583.
20. Ahn BJ, Kwon KY. Recurrent episodes of nocturnal hemiballism: A post-stroke movement disorder. *Parkinsonism & related disorders*. 2017;42:102-104. doi:10.1016/J.PARKRELDIS.2017.06.002
21. Aiba Y, Sakakibara R, Ogata T, Tateno F. Hemichorea Neglect after Stroke. *Case reports in neurology*. 2021;13(1):50-52. doi:10.1159/000512266
22. Bacchin R, Macchione F, Cardellini D, et al. Levofloxacin-induced hemichorea-hemiballism in a patient with previous thalamic infarction. *Neurological sciences : official journal of the Italian Neurological Society and of the Italian Society of Clinical Neurophysiology*. 2018;39(8):1483-1485. doi:10.1007/S10072-018-3298-1
23. Bembenek JP, Bilik M, Członkowska A. Successful treatment with intravenous recombinant tissue plasminogen activator in an acute stroke patient presenting with hemiballism. *Functional neurology*. 2015;30(1):71-72. doi:10.11138/FNeur/2015.30.1.071
24. Bertamino M, Signa S, Vagelli G, et al. An atypical case of post-varicella stroke in a child presenting with hemichorea followed by late-onset inflammatory focal cerebral arteriopathy. *Quantitative imaging in medicine and surgery*. 2021;11(1):463-471. doi:10.21037/QIMS-20-628
25. Carbayo Á, Sarto J, Santana D, Compta Y, Urra X. Hemichorea as Presentation of Acute Cortical Ischemic Stroke. Case Series and Review of the Literature. *Journal of stroke and cerebrovascular diseases : the official journal of National Stroke Association*. 2020;29(10). doi:10.1016/J.JSTROKECEREBROVASDIS.2020.105150
26. Chen H, Xu Z. Hemichorea-hemiballismus as the initial manifestation of symptomatic middle cerebral artery dissection: A case report. *Medicine*. 2020;99(36):e22116. doi:10.1097/MD.0000000000022116
27. Cotroneo M, Ciacciarelli A, Cosenza D, et al. Hemiballism: Unusual clinical manifestation in three patients with frontoparietal infarct. *Clinical neurology and neurosurgery*. 2020;188. doi:10.1016/J.CLINEURO.2019.105612

28. Damani A, Ghoshal A, Salins N, Deodhar J, Muckaden MA. Management of hemichorea hemiballismus syndrome in an acute palliative care setting. *Indian journal of palliative care*. 2015;21(1):72-75. doi:10.4103/0973-1075.150193
29. Disserol C, Alencar B, Parmera J, Conforto AB, Pinto LF. Teaching Video NeuroImages: Acute hemichorea-hemiballism reverted after IV thrombolysis. *Neurology*. 2020;94(1):e121-e122. doi:10.1212/WNL.0000000000008706
30. Ganapa S V., Ramani MD, Ebunlomo OO, Rahman RK, Herschman Y, Mammis A. Treatment of Persistent Hemiballism with Deep Brain Stimulation of the Globus Pallidus Internus: Case Report and Literature Review. *World neurosurgery*. 2019;132:368-370. doi:10.1016/J.WNEU.2019.08.247
31. Grassl N, Ebert AD, Szabo K, Aghazadeh Y, Platten M, Alonso A. Pearls & Oy-sters: Hemiballism and Orbitofrontal-like Syndrome in a Patient With Unilateral Tuberothalamic Stroke. *Neurology*. 2022;99(14):10.1212/WNL.0000000000201066. doi:10.1212/WNL.0000000000201066
32. Hernandez Fustes OJ, Puppi Munhoz R, Arteaga Rodriguez C, Hernandez Fustes OJ. Chorea as the First Manifestation of Cerebral Infarction. *Cureus*. 2020;12(3). doi:10.7759/CUREUS.7384
33. Hodde M, Rowe KE, Surapaneni K, Terrigno P, Brighenti A, Altschuler EL. Management of Severe Hemiballismus: Treatment Challenges in the Acute Inpatient Rehabilitation Setting: A Case Presentation. *PM & R : the journal of injury, function, and rehabilitation*. 2017;9(7):732-735. doi:10.1016/J.PMRJ.2016.10.023
34. Huang H, Goh SH. Acute Hemiballismus as the Initial Manifestation of Ischemic Stroke: A Case Report. *Clinical practice and cases in emergency medicine*. 2021;5(3):350-352. doi:10.5811/CPCEM.2021.5.52678
35. Kannepalli NRVL, Yadav R, Vazhayil V, Somanna S, Pal PK. Ipsilateral Hemichorea-hemiballism in a Case of Postoperative Stroke. *Tremor and other hyperkinetic movements (New York, NY)*. 2016;6. doi:10.7916/D8C53KQR
36. Kato H, Murakami T, Tajiri Y, Yamaguchi N, Ugawa Y, Hanajima R. [Hemichorea in a patient with acute cerebral infarction of the somatosensory cortex]. *Rinsho shinkeigaku = Clinical neurology*. 2021;61(5):325-328. doi:10.5692/CLINICALNEUROL.CN-001527
37. Kurihara S, Sato T, Murakami H, et al. Crossed Choreoathetosis Caused by Unilateral Thalamic Hemorrhage. *Journal of stroke and cerebrovascular diseases : the official journal of National Stroke Association*. 2021;30(10). doi:10.1016/J.JSTROKECEREBROVASDIS.2021.106049
38. Li ZS, Fang JJ, Xiang XH, Zhao GH. Hemichorea due to ipsilateral thalamic infarction: A case report. *World journal of clinical cases*. 2021;9(19):5287-5293. doi:10.12998/WJCC.V9.I19.5287
39. Maruyama K, Suzuki A, Mochizuki H, Shiomi K, Nakazato M. [A case of hemichorea due to insular and parietal cortical infarctions]. *Rinsho shinkeigaku = Clinical neurology*. 2021;61(7):491-493. doi:10.5692/CLINICALNEUROL.CN-001541
40. Miller NM, Alexander TC, Smith AT. Hemiballismus: An Activating Lesion. *The Journal of emergency medicine*. 2018;55(6):e153-e155. doi:10.1016/J.JEMERMED.2018.09.039
41. Miura S, Ueno M, Miyata R, Maniwa E, Etoh S, Shimodozono M. Mirror therapy for involuntary movement due to chronic thalamic hemorrhage: a case report. *European*

journal of physical and rehabilitation medicine. 2017;53(6):968-971. doi:10.23736/S1973-9087.17.04627-5
42. Motolese F, Pezzella FR, Marano M, Di Lazzaro V, Anticoli S. Post-stroke hyperkinetic movement disorders: a brain network issue. *Neurological sciences : official journal of the Italian Neurological Society and of the Italian Society of Clinical Neurophysiology*. 2021;42(4):1579-1581. doi:10.1007/S10072-020-04832-5
43. Muguruma K, Motoda A, Sugimoto T, Kitamura T. [A case of hemichorea caused by right internal carotid artery stenosis]. *Rinsho shinkeigaku = Clinical neurology*. 2019;59(8):509-514. doi:10.5692/CLINICALNEUROL.CN-001276
44. Muhammad A, Bielskute E, Guyler PC, Grunwald IQ. Acute ischaemic stroke-related choreoathetosis treated with arterial thrombectomy. *BMJ case reports*. 2019;12(1). doi:10.1136/BCR-2018-226035
45. Noda K, Hattori N, Okuma Y. Hemiballism with leg predominance caused by contralateral subthalamic haemorrhage. *BMJ case reports*. 2015;2015. doi:10.1136/BCR-2014-208525
46. Nohria R, Bennett S, O'Keefe YA. Haemiballism/haemichorea: an atypical presentation of ischaemic stroke. *BMJ case reports*. 2021;14(6). doi:10.1136/BCR-2020-240439
47. Onder H. Hemichorea-hemiballismus in the setting of posterolateral putaminal lesion and treatment with topiramate. *Journal of the neurological sciences*. 2017;375:388-389. doi:10.1016/J.JNS.2017.02.031
48. Onder H. Repeating Acute-Onset Hemiballismus in Association with Distinct Stroke Regions in a Single Patient. *Annals of Indian Academy of Neurology*. 2019;22(2):255-257. doi:10.4103/AIAN.AIAN_282_18
49. Qiu J, Cui Y, Sun L, Guo Y, Zhu Z. Hemichorea associated with cavernous angioma and a small errhysis: A case report and literature review. *Medicine*. 2018;97(43). doi:10.1097/MD.0000000000012889
50. Shahali H, Farahani RH, Asgari A, Hazrati E. Thalamic hemi-chorea: a rare complication after receiving the adenoviral vector-based COVID-19 vaccine: a case report. *Clinical and experimental vaccine research*. 2022;11(2):217-221. doi:10.7774/CEVR.2022.11.2.217
51. Shiraiwa N, Hoshino S, Saito G, Tamaoka A, Ohkoshi N. Clinical features of hemichoreahemiballism: A stroke-related movement disorder. *Neurology international*. 2020;12(1):6-8. doi:10.4081/NI.2020.8328
52. Shrestha P, Adhikari J, Poudel D, Pathak R, Karmacharya P. Cortical Hemiballism: A Case of Hemiballismus Associated with Parietal Lobe Infarct. *North American journal of medical sciences*. 2015;7(12):572-574. doi:10.4103/1947-2714.172850
53. Sikri V, Jain A, Singhal V, Gupta A. A rare case of movement disorder in Intensive Care Unit. *Indian journal of critical care medicine : peer-reviewed, official publication of Indian Society of Critical Care Medicine*. 2016;20(10):605-607. doi:10.4103/0972-5229.192055
54. Strauss S, Rafie D, Nimma A, Romero R, Hanna PA. Pure Cortical Stroke Causing Hemichorea-Hemiballismus. *Journal of stroke and cerebrovascular diseases : the official journal of National Stroke Association*. 2019;28(10). doi:10.1016/J.JSTROKECEREBROVASDIS.2019.07.003
55. Tatum PS, Kornbluth J, Soroka A. Embolic MCA Stroke Treated With tPA and TICI-3 Mechanical Thrombectomy Complicated by Hemiballismus: A Case Study and

Literature Review. *Military medicine*. Published online November 2021. doi:10.1093/MILMED/USAB436
56. Tolla AS, Schichtel LT, Alboudi A, Sanders AK. Anterior choroidal artery infarct presenting as right hemichoreoathetosis. *BMJ case reports*. 2020;13(5). doi:10.1136/BCR-2020-234861
57. Ueta Y, Kato H, Naito M, Taguchi T, Terashi H, Aizawa H. Persistent Hemichorea as a Preceding Symptom of Cerebral Infarction Due to Middle Cerebral Artery Stenosis. *Internal medicine (Tokyo, Japan)*. 2021;60(23):3805-3808. doi:10.2169/INTERNALMEDICINE.7191-21
